# New HfNbTaTiZr High-Entropy Alloy Coatings Produced by Electrospark Deposition with High Corrosion Resistance

**DOI:** 10.3390/ma14154333

**Published:** 2021-08-03

**Authors:** Ciprian Alexandru Manea, Mirela Sohaciu, Radu Stefănoiu, Mircea Ionuț Petrescu, Magdalena Valentina Lungu, Ioana Csaki

**Affiliations:** 1 Material Science and Engineering Faculty, University Politehnica of Bucharest, 060042 Bucharest, Romania; ciprian6@gmail.com (C.A.M.); radu.stefanoiu@upb.ro (R.S.); ionut.petrescu@upb.ro (M.I.P.); ioana.apostolescu@upb.ro (I.C.); 2 Metallic, Composite and Polymeric Materials Department, National Institute for Research and Development in Electrical Engineering ICPE-CA, 030138 Bucharest, Romania; magdalena.lungu@icpe-ca.ro

**Keywords:** high-entropy alloy, mechanical alloying, corrosion resistance

## Abstract

The aim of the present paper is to investigate an innovative high corrosion resistance coating realized by electrospark deposition. The coating material was fabricated from HfNbTaTiZr high-entropy alloy. HEA was produced by the mechanical alloying of Hf, Nb, Ta, Ti, and Zr high-purity powders in a planetary ball mill, achieving a good homogenization and a high alloying degree, followed by spark plasma sintering consolidation. The electrodes for electrospark deposition were cut and machined from the bulk material. Stainless steel specimens were coated and electrochemically tested for corrosion resistance in a 3.5% NaCl saline solution.

## 1. Introduction

HEAs are systems of alloys consisting of at least five elements in equal or almost equal atomic proportions. The large number of alloying elements leads to the formation of disordered solid solutions that maximize the configurational entropy. The microscopic analyses performed on HEAs may indicate ordered or disordered solid solutions, nanoprecipitates, or amorphous phases [1]. Senkov et al. [2,3] reported that the process of producing equiatomic TaNbHfZrTi alloy consisted of melting in a VAR furnace, followed by three remeltings of the material for a better homogenization. After that, the material was hot-isostatic-pressed at 207 MPa for 1 h at 1200 °C, followed by vacuum annealing for 24 h at 1200 °C.

This study investigates the opportunity to use an innovative high-entropy alloy produced by mechanical alloying to be used as coating for corrosion-resistant protection.

HEA properties could be designed depending on the added constituent elements, and in this way, new alloys with properties superior to classic alloys can be obtained. Among these properties, resistance to high temperature, wear, or corrosion is most interesting in this study.

High-entropy alloys could be produced either in liquid state, as the production method is represented by liquid-state processing in vacuum arc melting, or through induction melting furnaces [4,5,6,7].

Solid-state processing is represented by mechanical alloying and is used when the constitutive elements have melting temperatures with big differences and can exceed the evaporation temperature of other elements or when they are immiscible in liquid state [8,9,10].

Another advantage brought by mechanical alloying is the obtaining of a better homogeneity in cases of certain element combinations [11].

Using powder metallurgy results in the production of homogeneous materials at the molecular level, avoiding segregations or undesirable dendritic structures, which in general is a characteristic of the liquid state processing.

Due to the high cost of HEA, an economically efficient method is to coat with this type of alloys. The electrospark deposition (ESD) technique does not induce thermal stress to coated parts, and it can be used also for local repairs of the surface or to improve it. This technique is similar to the microwelding process, where successive layers are deposited by a manual or automatic process. During the process, an argon atmosphere is evacuated through a nozzle, creating a protective shield over the whole surface and a rapid cooling of the coating. With this technique, high-purity coatings are produced with a low oxygen concentration in the chemical composition due to the presence of the argon atmosphere during the deposition process [12,13].

HEA coatings can enhance resistance to high-temperature (thermal barriers) wear or corrosion without a major impact on the weight, geometry, or production cost of the part.

This paper’s aim is to obtain HfNbTaTiZr high-entropy alloy coatings by electrospark deposition designed for aggressive environments, where corrosion has a major negative impact on metallic materials, causing damage and even failure, which occurs due to the interaction between the material and the environment. Analyses of these coatings will provide information regarding the corrosion resistance of the coating applied on a stainless steel substrate. The material was produced by mechanical alloying, consolidated by spark plasma sintering, and then machined in an electrode suitable for electrospark deposition. The technique employed, electrospark deposition, allowed us to produce a thin layer, and thus the cost of this alloy could be reduced. For this paper, successive layers were deposited by the electrospark deposition technique in order to obtain a compact coating suitable for aggressive environments.

## 2. Materials and Methods

HfNbTaTiZr is a high-entropy alloy, and according to a calculated VEC value of 4.4, it has a body-centered cubic (BCC) structure. The alloy was developed by mechanical alloying from high-purity metallic powders in a Pulverisette 6 classic line planetary ball mill (Fritsch^®^, Idar-Oberstein, Germany) with a stainless steel vial and grinding media.

Due to the powder reactivity, handling and alloying were performed under an argon atmosphere. To avoid the welding of the alloy to the vial and balls and to reduce the oxidation and contamination of powders, n-heptane was used as a control process agent (PCA).

Milling parameters were selected after multiple trials to ensure that the powders were not overheating. A mixture of Hf, Nb, Ta, Ti, and Zr high-purity powders was milled for 60 h, and samples were taken at different time intervals to evaluate the alloying degree. After the alloying process, the particle size was reduced, and a good homogeneity was obtained. With further processing represented by sintering and deposition, the alloying degree was increased.

After the HfNbTaTiZr HEA development, the resulting powder was sintered by using the spark plasma sintering (SPS) technique with an HP D25 (FCT Systeme GmbH, Rauenstein, Germany) equipment in a vacuum (100 hPa) at a temperature of 1000 °C and a pressure of 50 MPa. The resulting samples had a density of 8.96 g/cm^3^, which represents approximately 90% from a theoretical density of 9.9 g/cm^3^, but also a uniform, homogeneous metallic appearance with no visible cracks or other defects.

One of the resulting samples was cut and machined in order to produce electrodes for the electro spark deposition process.

The HfNbTaTiZr HEA coating was obtained by performing successive passes on the substrate, changing the deposition direction after each pass, by using the Spark Depo Model 3 equipment (TechnoCoat International Co., LTD, Shizuoka, Japan) with a miniature applicator. The deposition parameters are presented in Table 1.

In order to avoid oxidation of the newly deposited coating, electrodeposition was performed under an inert gas shield; in this case, argon was used. The substrate was prepared in advance by sandblasting and alcohol decontamination. After sandblasting, the substrate had a rough and oxide-free surface, in this way, enhancing the adherence of the coating.

The coated sample surface and transversal section was investigated using an FEI Philips XL30 Environmental Scanning Electron Microscope with EDAX Sapphire energy dispersive spectrometry (EDS, SEMTech Solutions, Inc., North Billerica, MA, USA).

The Vickers hardness was measured with a Shimadzu Vickers hardness device (Shimadzu, Columbia, NY, USA) with 0.98 N load for the bulk sintered sample and for the deposited coating of HfNbTaTiZr high-entropy alloy along the surfaces with 10 different points, and the mean value was calculated. In order to observe the coating effect on the substrate, measurements were performed on the stainless steel substrate under the same conditions.

The coated sample was electrochemically tested in an electrochemical cell with saline solution (3.5% NaCl) at room temperature to monitor the corrosion behavior of HEA by using a galvanostat/potentiostat model PARSTAT 4000, an Ametek equipment (Princeton Applied Research—Ametek, Oak Ridge, TN, USA)) with an SCE (saturated calomel electrode) as a reference electrode, a platinum electrode for recording, and the sample as a working electrode.

The corrosion resistance was tested according to the ASTM G5-94(2011) standard [14]. The open circuit potential (E_oc_) was measured for 6 h, and the polarization potentiodynamic Tafel curves were plotted from −0.2 to +0.2 V with a scanning rate of 1 mV/s.

## 3. Results

The composition of the HEA alloy employed in this work was designed according to theoretical calculations of the solid solution formation parameter (Ω), entropy of mixing *(*Δ*S_mix_),* enthalpy of mixing (Δ*H_mix_*), atomic size difference (δ), and valence electron concentration (*VEC*) [15,16].

(1) $$\Omega =\frac{T_{m}\Delta S_{mix}}{\Delta H_{mix}}$$

(2) $$\Delta S_{mix}=-R\overset{n}{\underset{i=1}{\sum }}c_{i}lnc_{i}$$

(3) $$\Delta H_{mix}=\overset{n}{\underset{i=1,\;i\neq j}{\sum }}4\Delta H_{ij}^{mix}c_{i}c_{j}$$

(4) δ=∑i=1nci1−rir¯

(5) $$VEC=\overset{n}{\underset{i=1}{\sum }}c_{i}VEC_{i}$$

where *n* is the number of components, *i* and *j* refer to a specific component, *c* is the atomic fraction, *T_m_* is the melting point of the alloy (defined by the mixing rule), *R* is the ideal gas constant, $\Delta H_{ij}^{mix}$ is the mixing enthalpy of binary alloys in liquid state, *r* is the atomic radius, and $\bar{r}=\Sigma c_{i}r_{i}$ is the average radius of the alloy.

According to Zhang et al. [17], the phase stability formation criteria are fulfilled when Ω ≥ 1.1 and δ ≤ 6.6, a solid solution being formed. Takeuchi et al. [18] studied the effects of the mixing entropy changes on the formation of the crystalline structure. The authors found that high mixing entropy of HEAs could overcome the enthalpy of intermetallic compound formation. Additionally, the valence electron concentration (*VEC*) seems to play a decisive role in determining the crystal structure solid solution forms in HEAs, face-centered cubic (FCC) or body-centered cubic (BCC). According to Guo et al. [19], larger *VEC* (≥8) favors the formation of FCC-type solid solutions, while smaller VEC (<6.87) favors the formation of BCC-type solid solutions.

The thermodynamic parameters for the HfNbTaTiZr alloy are calculated according to Equations (1)–(5) and are reported in Table 2.

A study published by Parakha et al. [20] examined the correlation between the crystal structure and the corrosion resistance properties of AlCoCrFeNi high-entropy alloy, and the results present that by increasing the FCC content, the alloy is more susceptible to corrosion.

The produced HEA was examined by electron microscopy. The investigated samples are from the powder obtained by mechanical alloying, and from the sintered material that was transformed into electrode for the electro spark deposition process for the coating obtained. 

**Figure 1:**
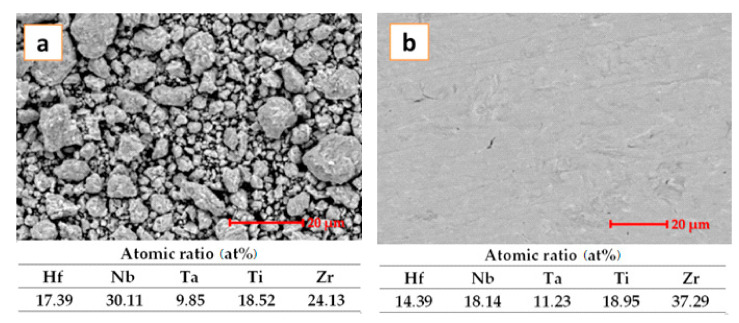
SEM images and EDS analyses of (**a**) mechanically alloyed powder and (**b**) sintered HEA.

The investigation was realized for each stage to observe the transformation occurring during HEA processing. The mechanical alloying process was widely described in a previous paper [21], where the following parameters were used: 300 RPM speed for 30 h in an argon atmosphere with 10:1 BPR and n-heptane as PCA. The process was further improved by increasing the milling time up to 60 h. The mean particle size dimension of the initial pure powders was 63 μm. The microstructure of the sample is presented in Figure 1a. The morphology shows a homogeneous character of the mechanically alloyed sample with all elements present, as the EDS analyses revealed. The microstructure of the SPSed HEA alloy is presented in Figure 1b. The image reveals a compact structure with no cracks or voids.

The electrode was machined from the sintered part. In order to obtain the ideal dimension for the electrode, the middle section of the sample was selected. The shape was formed for a better grip with the applicator holder, and the tip had a cone shape, which favors the deposition process, by increasing the contact surface and the deposition angle.

**Figure 2:**
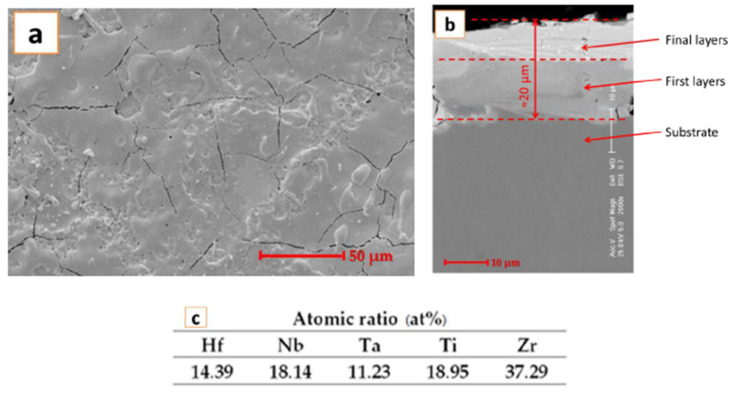
SEM images and EDS analysis results for the HfNbTaTiZr high-entropy alloy coating deposited by the ESD technique for (**a**) top view and (**b**) cross section; (**c**) EDS analyses results.

The coating realized with the electrode fabricated in our laboratory was also investigated with a scanning electron microscope. The SEM images are presented in Figure 2.

For the surface analysis results presented in Figure 2a, it can be observed that due to this type of deposition, fine fissures are present. The fissures are formed due to the rapid cooling of the deposited layer on the substrate. After the sample was imbedded and cut, the cross section was analyzed, and the results are presented in Figure 2b. It can be observed that a thickness of approximately 20 μm was obtained, and the deposited layer was compact after the successive passes. The fissures were not present at the interface between the substrate and the deposited layer, which indicates good adhesion. The adhesion testing was previously performed [22], and the results confirm the obtained layer performances.

EDS analysis results, presented in Figure 2c, confirm the layer composition, where no contamination is present.

**Figure 3:**
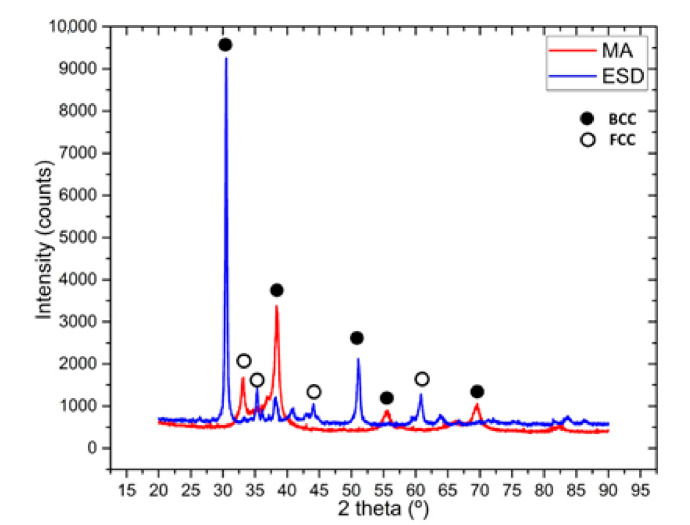
XRD analysis results for the mechanically alloyed HfNbTaTiZr high-entropy alloy after 60 h of milling time and the electrospark deposited sample.

The XRD pattern, presented in Figure 3, reveals that the main phase is bcc. The peaks after mechanical alloying reveals a good alloying degree with the main BCC phase confirmed also in this alloy’s VEC. We previously investigated this alloy, and the alloying degree was low after 30 h of mechanical alloying [21]. However, now the XRD revealed a good alloying degree, and we could use the powder to further process and manufacture the ESD electrode. The coating realized with the electrode manufactured like this reveals the main BCC peaks and some very small FCC peaks, which are probably present due to the substrate influence.

A good investigation method for evaluating the improvement of the mechanical properties could be the hardness measurement. The coated sample’s hardness was measured in order to evaluate the coating behavior on the HfNbTaTiZr HEA layer. The hardness of the consolidated electrode was measured as well, and it was compared with the hardness of the 316 stainless steel substrate. The measurement of the hardness samples was performed with the same device and using the same pattern for collecting 10 measurements for each sample. The results are listed in Table 3. The measurements were performed on the diagonals of the sample, with 5 measurements on each, and the mean value is presented.

The sample was subjected to corrosion in a saline environment. The coating was thin (approx. 20 μm), and the corrosion resistance could have been influenced by the coating thickness.

**Figure 4:**
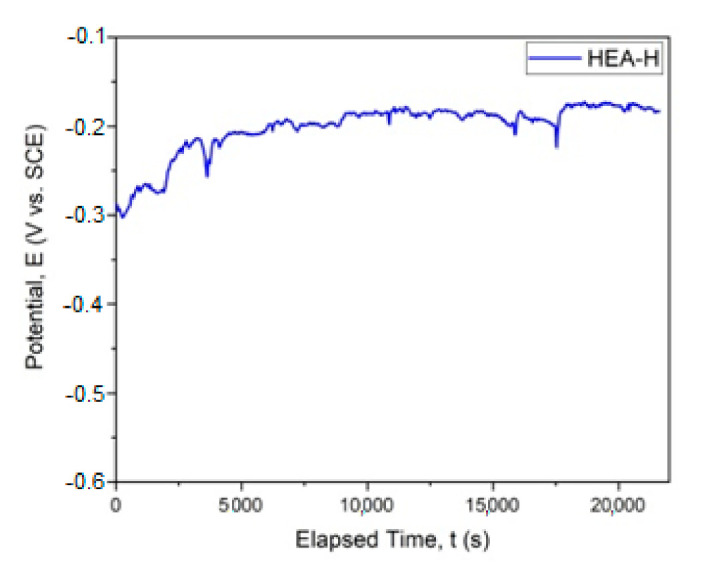
Corrosion potential for HfNbTaTiZr HEA. (HEA-H).

**Figure 5:**
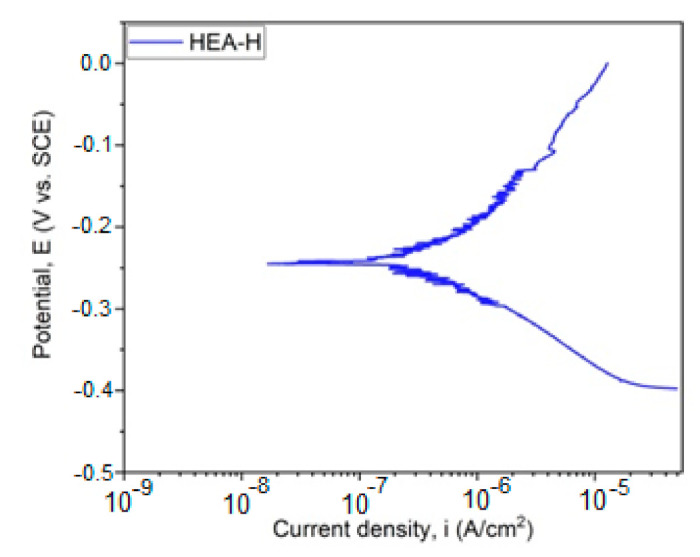
Tafel plot for HfNbTaTiZr HEA. (HEA-H).

The graphical representation of E_corr_ evolution is presented in Figure 4. In Figure 5, the Tafel plot is revealed.

With the aid of the Tafel parameters, the following parameter characteristics for corrosion resistance were determined: corrosion potential E_corr_, density of corrosion current I_corr_, cathodic slope *β_c_*, and anodic slope *β_a_*. With the aid of Tafel extrapolation, the polarization resistance was calculated in order to evaluate the corrosion resistance and the results are presented in Table 4. The polarization resistance was realized according to ASTM G59-97 (2014) [23] with the aid of the following equation:

(6) $$R_{p}=\frac{1}{2.3}\frac{\beta_{a}|\beta_{c}|}{\beta_{a}+\left|\beta_{c}\right|}\frac{1}{i_{cor}}\;$$

The corrosion rate was calculated according to ASTM G102-89 (2004) [24] with the following equation:

(7) $$CR=K_{i}\frac{i_{corr}}{\rho }EW$$

where *CR* is the corrosion rate (mm/year), *K_i_* is 3.27 × 10^−3^, ρ is the material density (g/cm^3^), *i_corr_* is the current density of the material (µA/cm^2^), and *EW* is the equivalent weight (g).

**Figure 6:**
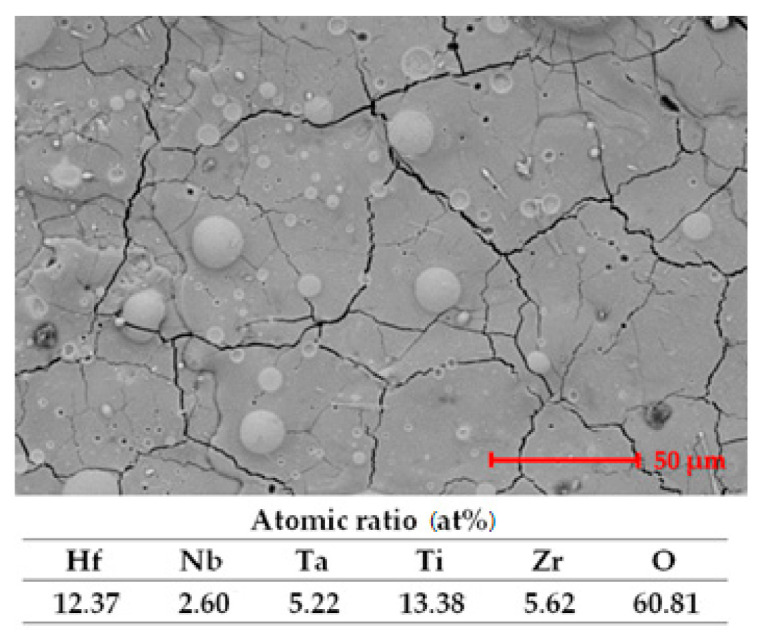
SEM image and EDS analysis results after exposure to corrosion.

In Figure 6, the microstructure and EDS analysis results are presented.

The figure presents some traces of the passivation film formed. The oxygen present in the EDS analysis results confirms the presence of the passivation film. The cracks seem filled in, but the SEM image is not very different in comparison with the microstructure presented in Figure 2.

## 4. Discussion

For this paper, it was decided that the highest calculated entropy value for the Hf, Nb, Ta, Ti, and Zr mixture is to be subjected to multiple studies. According to the thermodynamic calculations, the maximum values for δ, Δ*S_mix_,* and Δ*H_mix_* are achieved for the equiatomic HEA, which also fulfills the conditions [19] for solid solution formation. Increasing or decreasing the amount of tantalum leads to a decrease in the parameter values.

The promising results obtained after the mechanical alloying of the HfNbTaTiZr mixture led to the final goal of obtaining the corrosion-resistant coating of this material for aggressive environments. The cut and machined electrodes were subjected to tests and trials in order to obtain an efficient deposition and a homogeneous coating, where no cracks or defects were present, by adjusting the shape according to the miniature applicator holder but also the electrospark parameters. The deposition temperature is an important factor for this deposition technique due to the increased probability of crack and defect formation.

The deposited coating of HfNbTaTiZr was SEM-surface-analyzed, and the results present a homogeneous layer with fine fissures, which are common for this type of deposition. The samples were imbedded in resin and cut, and the SEM analysis results present a compact coating with a thickness of approximately 20 µm. The composition is confirmed by the EDS analyses, where no contamination is present after the multiple-stage process. The microstructure evolution from powder to coating is very interesting. The consolidated sample has a very homogeneous distribution, all the elements are present, and there is no oxygen detected in the electrode microstructure. The coating presents some minor cracks on the surface coming from the rapid cooling of the electrospark deposition process. The cracks, however, are not present in the coating structure, as they can be observed in the cross section. Additionally, the corrosion results obtained prove that the small cracks did not influence the corrosion resistance of the coating despite its thickness.

The hardness tests performed on the bulk consolidated sample revealed an increased value of the hardness. Málek et al. [25] determined the HV hardness for mechanically alloyed spark-plasma-sintered TaTiHfNbZr high-entropy alloy, and the value was 584 HV. For spark plasma sintering processing, different parameters were used, achieving a consolidation degree of over 90%. Thus, the hardness value for the bulk SPS specimen was high, 840 HV. The small cracks appearing on the sample’s superficial layer were due to this high value. The coating layer was thin (approx. 20 μm), and there was only a small difference in the hardness value between the layer and the substrate.

Corrosion testing results, for the sample immersed in a saline environment, are promising, taking into consideration the thickness of the obtained coating. The results present a good electropositive value of corrosion potential and resistance to polarization, indicating a desired behavior for this type of coating in very corrosive media. The results obtained in this study can be compared with the values obtained from testing 316 stainless steel under similar conditions, obtaining a value for E_corr_ of −655.6 mV and a value for *i_corr_* of 12.53 µA/cm^2^ [26].

The calculated corrosion rate and the microstructure not affected by the corrosion reveals a very good corrosion rate despite the thin film applied and the minor cracks present on the top surface. In comparison with 316 stainless steel tested in the same way [26], the result was dramatically improved. This means that the coating could be used in a corrosive environment, increasing the lifetime of a coated component.

## 5. Conclusions

A high-entropy alloy was developed using raw pure powders of Hf, Nb, Ta, Ti, and Zr by mechanical alloying, followed by spark plasma sintering. The electrode for electro spark deposition was machined and used to coat a thin layer on a stainless steel sample.

The coating obtained was homogeneous, and the microstructure analyses revealed that even though fine fissures are present on the surface of the deposited sample, there are none present close to the substrate and coating interface. This results in a good adhesion and distribution of the coating over the substrate.

Hardness was measured on the bulk sample and also on the coating. The coating had an improved value of the hardness in comparison with the substrate, and the high value obtained for the bulk sample probably induced small cracks on the coated layer surface.

The corrosion rate obtained after a test in chlorine containing fluid was very small (0.00024 mm/year), and the results encourage us to go further with testing the samples in other chlorine-containing fluids as geothermal fluids. Future work will focus on the further optimization of the electrospark deposition parameters in order to reduce the cracking possibility. For an even better resistance to corrosion, thicker layers will be deposited and subjected to multiple aggressive media tests, including in situ in a geothermal environment.

## Figures and Tables

**Table 1:** ESD parameters for HfNbTaTiZr HEA coatings on stainless steel substrates.

Coating	Substrate	Capacitance (µF)	Voltage (V)	Frequency (Hz)	Gas Shield
HfNbTaTiZr	SS 316 L	40	100	260	Argon

**Table 2:** Calculated thermodynamic parameters for the HfNbTaTiZr HEA alloy.

Parameter	Units	Value
VEC	-	4.4
δ	-	4.99
ΔS_mix_	J/K mol	13.37
ΔH_mix_	kJ/mol	2.72
Ω	-	12.36

**Table 3:** Hardness mean value and the standard deviation for the tested samples.

Material	Hardness Mean Value (HV)	Standard Deviation
Substrate (316 SS)	250	27.56
HfNbTaTiZr HEA sintered	840	67.61
HfNbTaTiZr HEA coating	254	28.20

**Table 4:** Corrosion resistance parameters for the HfNbTaTiZr HEA alloy.

Sample	E_oc_ (mV)	E_cor_ (mV)	*i_cor_* (μA/cm^2^)	*β_c_* (mV)	*β_a_* (mV)	*Rp* (kΩxcm^2^)	*CR* (mm/year)
HEA-H	−183	−244	0.0056	97.68	174.06	48.84	0.00024

## Data Availability

Data sharing not applicable.
